# Feasibility of [^18^F]FSPG PET for Early Response Assessment to Combined Blockade of EGFR and Glutamine Metabolism in Wild-Type *KRAS* Colorectal Cancer

**DOI:** 10.3390/tomography9020041

**Published:** 2023-02-24

**Authors:** Seong-Woo Bae, Jianbo Wang, Dimitra K. Georgiou, Xiaoxia Wen, Allison S. Cohen, Ling Geng, Mohammed Noor Tantawy, H. Charles Manning

**Affiliations:** 1 Department of Cancer Systems Imaging, The University of Texas MD Anderson Cancer Center, 1515 Holcombe Blvd., Houston, TX 77030, USA; 2 Vanderbilt Center for Molecular Probes, Vanderbilt University Medical Center, 1161 21st Avenue South, Medical Center North, AA-1105, Nashville, TN 37232, USA; 3 Vanderbilt University Institute of Imaging Science, Vanderbilt University Medical Center, 1161 21st Avenue South, Medical Center North, AA-1105, Nashville, TN 37232, USA; 4 Department of Radiology and Radiological Sciences, Vanderbilt University Medical Center, 1161 21st Avenue South, Medical Center North, Nashville, TN 37232, USA

**Keywords:** PET, FSPG, glutaminolysis, EGFR, colorectal cancer

## Abstract

Early response assessment is critical for personalizing cancer therapy. Emerging therapeutic regimens with encouraging results in the wild-type (WT) *KRAS* colorectal cancer (CRC) setting include inhibitors of epidermal growth factor receptor (EGFR) and glutaminolysis. Towards predicting clinical outcome, this preclinical study evaluated non-invasive positron emission tomography (PET) with (4S)-4-(3-[^18^F]fluoropropyl)-L-glutamic acid ([^18^F]FSPG) in treatment-sensitive and treatment-resistant WT *KRAS* CRC patient-derived xenografts (PDXs). Tumor-bearing mice were imaged with [^18^F]FSPG PET before and one week following the initiation of treatment with either EGFR-targeted monoclonal antibody (mAb) therapy, glutaminase inhibitor therapy, or the combination. Imaging was correlated with tumor volume and histology. In PDX that responded to therapy, [^18^F]FSPG PET was significantly decreased from baseline at 1-week post-therapy, prior to changes in tumor volume. In contrast, [^18^F]FSPG PET was not decreased in non-responding PDX. These data suggest that [^18^F]FSPG PET may serve as an early metric of response to EGFR and glutaminase inhibition in the WT *KRAS* CRC setting.

## 1. Introduction

Colorectal cancer (CRC) is the third most common malignancy, the third leading cause of cancer-related deaths in men and in women, and the second most common cause of cancer deaths when numbers for men and women are combined [1]. Numerous drugs for patients with CRC have been developed and brought into preclinical and clinical trials, especially for metastatic colorectal cancer (mCRC), yet response rates remain limited [2]. For instance, the epidermal growth factor receptor (EGFR) neutralizing monoclonal antibodies cetuximab (an immunoglobulin G1 (IgG1) mouse–human chimeric monoclonal antibody) and panitumumab (a recombinant, fully humanized, IgG2 monoclonal anti-EGFR antibody) are both approved by the Food and Drug Administration (FDA) as standard of care for patients with CRC [3,4,5]. However, only 12–17% of patients with wild-type (WT) kirsten rat sarcoma virus (*KRAS)* have durable responses to anti-EGFR monotherapy [6]. Although response rates to EGFR-targeted therapies in patients with advanced WT *KRAS* CRC have been disappointing, combined blockade of EGFR and glutaminolysis has exhibited promising efficacy in preclinical studies [7], and is being currently explored clinically in CRC (NCT 03263429).

Biomarkers to predict response to glutaminase inhibitors, as well as EGFR inhibitors, remain a clinically unmet need. We hypothesized that positron emission tomography (PET) imaging of glutaminolysis represents a sensitive biomarker to predict early response to therapeutic regimens that include glutaminase inhibition. The ^18^F-labeled glutamate analog, (4S)-4-(3-[^18^F]fluoro-propyl)-l-glutamate ([^18^F)FSPG), is a promising tracer for PET imaging of tumors [8]. [^18^F]FSPG is taken up by cells through the cystine/glutamate transporter (xCT), which is commonly activated in numerous cancer types. In cancer models, [^18^F]FSPG has demonstrated the ability to predict drug-resistance by detecting elevated antioxidant pathways, preceding other markers such as tumor shrinkage and decreased glucose consumption [9,10]. We have previously evaluated [^18^F]FSPG PET as a diagnostic agent in hepatocellular carcinoma [11] and lung cancer [12].

Towards predicting clinical outcome in clinical trials, we hypothesized that [^18^F]FSPG PET could predict response to combined glutaminase and EGFR inhibition in WT *KRAS* CRC, a regimen currently being explored in a clinical trial (NCT 03263429). The aims of the current study were to evaluate [^18^F]FSPG uptake before and after treatment in WT *KRAS* CRC patient-derived xenograft (PDX) murine models of varying treatment sensitivity to explore the relationship between PET imaging and treatment response.

## 2. Materials and Methods

### 2.1. Sequencing Data Acquisition

Total RNA extract of each PDX tissue was digested by DnaseI (NEB, M0303S), purified by oligo-dT beads (Dynabeads mRNA purification kit, Invitrogen, 61011, Carlsbad, CA, USA), then poly(A)-containing mRNA were fragmented into 200–250 bp with fragment buffer (Ambion, AM8740, Austin, TX, USA). Double strand cDNA synthesis and (standard or strand-specific) sequencing libraries were prepared and validated following the sequencing provider’s RNA-Seq protocols. Sequencing was done using Illumina HiSeq-2000/2500/4000 and Novaseq 6000 in 100/126/150 bp paired-end (PE) reads with an expected throughput of 10G–18G bases per sample. Xenome, with an xenome-index built from GRCh38 and GRCm38, was utilized to classify reads from xenograft samples into human, mouse, both, ambiguous and neither. Only human reads were used for further downstream analyses.

PE reads were mapped to the respective reference genome (GRCh38 or GRCm38) using hisat2 (splice junction aware mapper using bowtie), resulting in a .bam file. Mapped reads were matched to GENCODE v27 and processed using stringtie to obtain raw counts and normalized gene expression values (COUNTS, FPKM, and TPM values). For further analyses using RNA expressions, we used raw counts to compute the corresponding expression values in Counts Per Million (CPM) using *edgeR* [13] with library sizes normalized by the Trimmed Mean of M-values (TMM).

Before variant calling, .bam files were re-preprocessed according to GATK best practices. First, duplicated reads were marked and removed using PicardTools; second, reads were split containing introns (N) in their compact idiosyncratic gapped alignment report (CIGAR) string using GATK SplitNCigarReads; and third, the mapping was recalibrated with GATK BaseRecalibrator and GATK ApplyBQSR. Furthermore, exon, transcript and gene coverage were calculated using BEDTOOLS on the re-preprocessed .bam files. Then variants were called as in the whole exome sequencing pipeline, using GATK haplotyper for variant detection, GATK VariantFiltration for filtering and VEP for variant annotation. The filtered variants were used to calculate the tumor mutational burden (number of somatic mutations per Mb sequenced).

### 2.2. In Vivo Tumor Studies

All animal procedures were approved by the Vanderbilt University Institutional Animal Care and Use Committee (IACUC). For in vivo studies, WT *KRAS* CRC PDX tissues (CXF233, CXF1784, CXF1972, and CXF1753), obtained from the Charles River PDX model repository, were inoculated in the right flank of 6-week-old female athymic nude mice. Mice were randomly assigned to treatment cohorts. Mice were treated with either phosphate-buffered saline (PBS) as vehicle control, αEGFR (cetuximab for CXF233 or panitumumab for CXF1784 and CXF1972), CB-839 (Calithera Biosciences, South San Francisco, CA, USA), or combined αEGFR and CB-839. Treatment was started when the tumor volume reached 100–200 mm^3^. αEGFR was administered at 1 mg/kg every 3 days for a total of 7 cycles. CB-839 was administered at 200 mg/kg orally every 12 h. Tumor volumes were measured manually using calipers every third day and quantified using the formula V = W * L * H/2.

### 2.3. Preclinical [^18^F]FSPG PET Imaging

[^18^F]FSPG was produced by radiolabeling of the protected precursor di-tert-butyl-(2S,4S)-2-(3-((naphthalen-2-ylsulfonyl)oxy)propyl)-4-(tritylamino)pentanedioate) with cyclotron-generated [^18^F]fluoride in the presence of K+- K2.2.2/K2CO3. After acidic deprotection, neutralization, and aqueous dilution, the tracer was purified over SPE cartridges and finally formulated for intravenous (i.v.) injection by passing the solution through a sterile filter. The synthesis was performed within an automated synthesis module (GE MX Reaction Module) in a lead-shielded hot cell. The production methods, sterile filtration, and formulation allowed for the production of a sterile and pyrogen-free solution ready for injection. A small aliquot was removed for analysis to confirm the quality of the final product solution.

The PET with computed tomography (CT) studies were carried out following an overnight fast in clean cages. Previously, we determined that forty minutes uptake was sufficient for [^18^F]FSPG accumulation to steady state in tumors (data not shown). The mice received a retro-orbital injection of ~9 MBq/0.1 mL of [^18^F]FSPG under anesthesia with 2% isoflurane and were returned to plate-warmed cages. Forty minutes later, the mice were anesthetized with 2% isofluorane and imaged with an Inveon microPET or Focus220 (Siemens Preclinical, Knoxville TN) for 10 min (Inveon) or 20 min (Focus220). Data from all possible lines of response (LOR) were saved in the list mode raw data format. The raw data were then binned into 3D sinograms with a span of 3 and ring difference of 47. The images were reconstructed into transaxial slices (128 × 128 × 95) with voxel sizes of 0.0475 × 0.0475 × 0.0796 cm^3^, using the MAP algorithm with 16 subsets, 4 iterations, and a beta of 0.0468. For anatomical co-registration, immediately following the PET scans, the mice received a CT scan in a NanoSPECT/CT (Mediso, Washington, DC, USA) at an X-ray beam intensity of 90 mAs and X-ray peak voltage of 45 kVp. The CT images were reconstructed into 170 × 170 × 186 voxels at a voxel size of 0.4 × 0.4 × 0.4 mm^3^. The PET/CT images were converted into DICOM files and were uploaded into OsiriX lite (Pixmeo, Bernex, Switzerland). The volumes of interest (VOIs) were drawn around the tumors. [^18^F]FSPG uptake as determined by the PET images was normalized to the injected dose and the mean radiotracer concentration within the VOIs.

The change in [^18^F]FSPG uptake (%) comparing pre- and post-treatment data was calculated as follows:

$$\mathrm{Uptake}\;\mathrm{change}\;\left(\%\right)=100\times \frac{{\mathrm{Uptake}}_{\mathrm{Post}-\mathrm{treatment}}-{\mathrm{Uptake}}_{\mathrm{Pre}-\mathrm{treatment}}}{{\mathrm{Uptake}}_{\mathrm{Pre}-\mathrm{treatment}}}$$

All results are represented as mean ± standard deviation (SD).

### 2.4. Histological Evaluation

Immediately after sacrifice, harvested PDX tissues were fixed in 10% formaldehyde for 24 h, dehydrated in 70% to 100% series ethyl alcohol, cleared in xylene and embedded in paraffin. Tissue blocks were cut to 5 μm. Paraffin sections were deparaffinized and rehydrated with distilled water and then stained with hematoxylin and eosin (H&E)**.** The stained sections were scanned into eSlideManager using scanscope. Images were analyzed using Halo v3.5.3577.140. Software (Indica Labs). Whole nodules (tumor area) were annotated, and the areas of alive tumor, fibrosis, necrosis and blank (no tissue) were classified and measured using determinate classifiers.

The normalized tumor area was calculated as follows:

$$\mathrm{Normalized}\;\mathrm{tumor}\;\mathrm{area}\;=\frac{{\mathrm{Whole}\;\mathrm{nodule}}_{ij}}{\mathrm{mean}\;\left({\mathrm{Whole}\;\mathrm{nodules}}_{\mathrm{Vehicle}}\right)}$$

where i and j stand for each sample (*n* = 5) and each group (vehicle or CB-839 + αEGFR), respectively.

### 2.5. Immunohistochemistry (IHC)

Paraffin sections of CRC PDX tissues were deparaffinized and hydrated. Heat-mediated antigen retrieval was performed with Tris-EDTA buffer (pH 9.0). Blocking of endogenous peroxidase was accomplished by incubation with 0.3% H_2_O_2_ in methanol for 15 min. Hydrated sections were incubated in 2.5% normal horse serum at room temperature for 1 h for blocking non-specific IgG. Sections were incubated with primary antibody rabbit polyclonal anti-SLC7A11/xCT (1:200; 26864-1-AP; Proteintech, Rosemont, IL, USA) or rabbit monoclonal anti-CD44 (1:200; #37259; Cell Signaling, Danvers, MA, USA) at room temperature for 1.5 h. After washing with PBS, sections were incubated with ready to use Biotinylated Pan-Specific Universal Antibody (PK-7800, Vector Laboratories, Burlingame, CA, USA) for 10 min at room temperature and Streptavidin/Peroxidase for 5 min at room temperature. After washing with PBS, sections were incubated with peroxidase substrate (ImmPACT®DAB, SK-4105, Vector Laboratories) at room temperature for 2 min. Harris’s modified hematoxylin was used for nucleus counterstaining. Sections were dehydrated through series of 70% to 100% alcohol, cleared in xylene and mounted with paramount and coverslipped. Images were captured by using LEICA DFC425 camera and LAS V3.7 software. IHC slides were read by an experienced tissue morphologist. Five areas in an IHC stained slide were randomly chosen under 10× magnification, then counted.

### 2.6. Statistical Analysis

All statistical analyses and graphs were generated with R (version 4.2.2) or GraphPad Prism 9. Unpaired *t*-test was used to determine the statistical significance of treatment evaluation or the [^18^F]FSPG uptake change between pre- and post-treatment or histologic evaluation.

## 3. Results

### 3.1. Characterization of Wild-Type KRAS PDX Tumors

**Figure 1:**
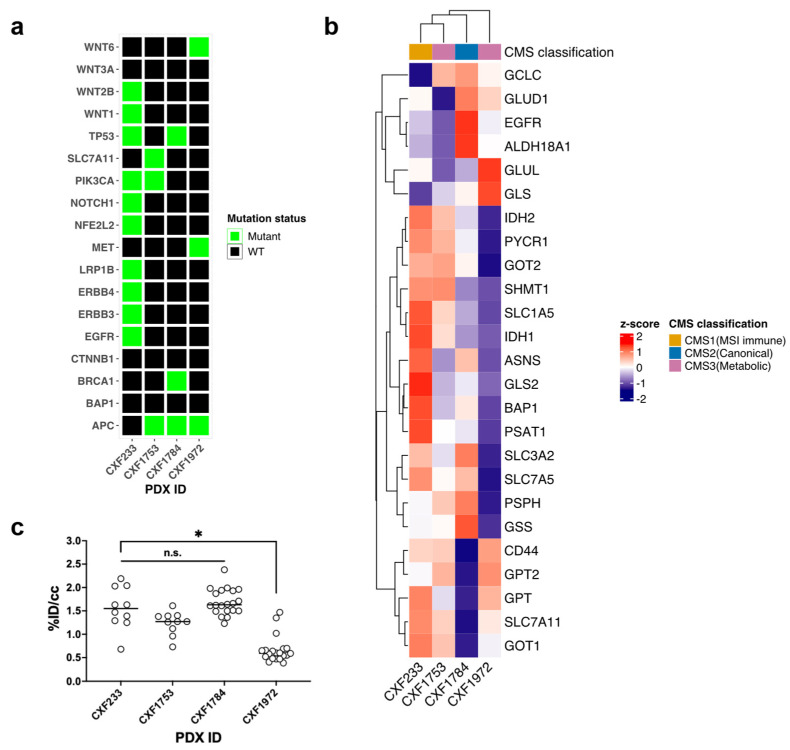
Baseline characteristics of wild-type (WT) KRAS colorectal cancer (CRC) patient-derived xenografts (PDXs). (**a**) Select mutational status highlighted in green for each PDX. (**b**) Transcriptomic profile with glutaminolysis-related genes. The consensus molecular subtype (CMS) was identified based on gene expressions of CRC. (**c**) Baseline (4S)−4−(3−[^18^F]fluoro−propyl)−l−glutamate ([^18^F)FSPG) uptake in PDX tumors. Each dot indicates [^18^F]FSPG uptake in a PDX tumor of a mouse. [^18^F]FSPG uptake as measured by the PET images were normalized to the injected dose and the mean radiotracer con-centration within the volumes of interest (VOIs) drawn around the tumors. n.s.: “not significant”, *: *p* < 0.0001.

We initially characterized the baseline features of PDXs derived from four different patients with CRC. The clinicopathological features of the donor patients for the PDX tissues used in mouse models are summarized in Table 1. Mutation status of each PDX was detected by whole exome sequencing (Figure 1a). The four PDXs evaluated exhibited a diversity of mutational characteristics by design; while all the PDX expressed WT KRAS, CXF233 exhibited a higher number of potentially relevant mutations including EGFR, PIK3CA, certain WNT genes, and multiple ERB family genes. Other cases exhibited relatively fewer mutations. Transcriptomic profiling of glutaminolysis-related genes showed distinct RNA expression levels across metabolic machinery and represented three consensus molecular subtypes (CMS) [14], classified as CMS1 (hypermutation and immune signature), CMS2 (canonical subtype with WNT and MYC activation), CMS3 (metabolic subtype) (Figure 1b). Heatmap clustering of select differentially expressed genes demonstrated that CXF233 and CXF1753 exhibited similar gene glutaminolysis-related expression based on the correlation distance defined as (1-Pearson) [15]. As expected, baseline [^18^F]FSPG uptake in CXF1972 tumors was modest compared to the others given its lower expression of many glutaminolysis-related genes (Figure 1c, *p* < 0.0001). Based on the unique RNA signatures, coupled with the baseline [^18^F]FSPG uptake, we initially selected CXF233, CXF1784, and CXF1972 for subsequent investigation.

### 3.2. In Vivo Evaluation of Treatment

**Figure 2:**
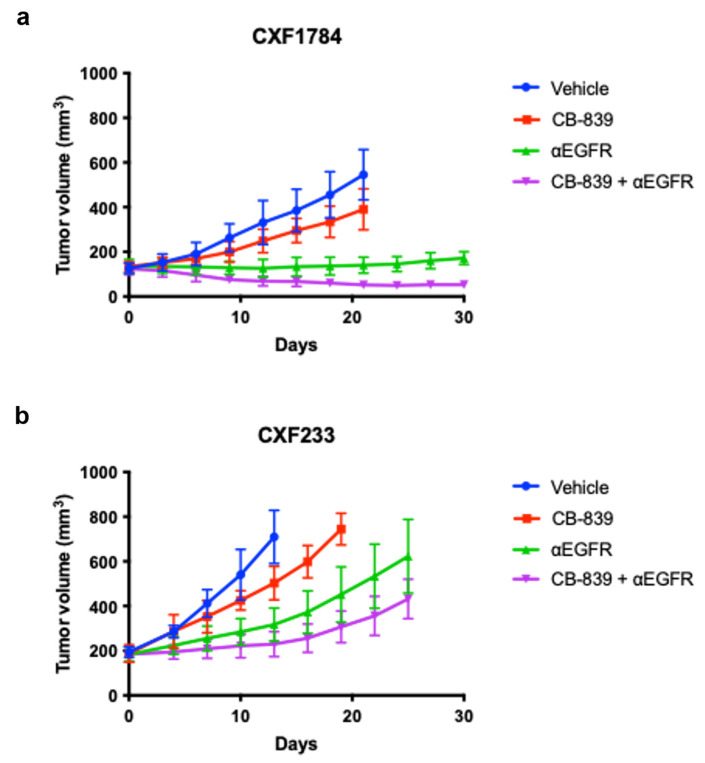
Treatment of WT KRAS PDX with a glutaminase inhibitor, 𝛼EGFR mAb, or the combination. PDX murine models were treated with either αEGFR monoclonal antibody (mAb), CB-839, or CB-839 and αEGFR. Error bars represent ± standard deviation (SD) (*n* = 5). (**a**) CXF1784 PDX was responsive to αEGFR or the combined therapy. (**b**) CXF233 PDX lacked response beyond growth delay with αEGFR or the combined therapy.

Three different WT KRAS CRC PDX tumors representing the three CMS sub-types were treated with either vehicle (PBS), a small molecule glutaminase inhibitor (CB−839), an EGFR-neutralizing monoclonal antibody (αEGFR), or a combination. Tumor volume was regularly monitored. As expected, the CXF1784 case (Figure 2a) progressed with vehicle treatment. Tumor growth was only moderately slowed by single agent CB-839 (*p* = 0.04). However, there were significant reductions in tumor growth for αEGFR mAb treatment (*p* < 0.0001) and the combined treatment (*p* < 0.0001) group compared to vehicle group. The combined treatment showed higher efficacy in comparison to the αEGFR mAb treatment group from day 21 post-treatment (*p* = 0.0009) through day 30 (*p* < 0.0001). Similarly, the CXF1972 case (Figure S1a) progressed with vehicle and single agent CB-839 treatment, while tumor growth in the αEGFR mAb treatment (*p* = 0.04) and the combined treatment group (*p* = 0.02) were slightly reduced. There was no statistical difference in efficacy between αEGFR mAb treatment group and the combined treatment group (*p* = n.s.). All treatments, either single agent or combination, led to progression in the CXF233 case (Figure 2b). Similar to vehicle treatment, tumor growth with single agent CB-839, αEGFR mAb, or the combination was modestly delayed. Overall, we found CXF1784 to be sensitive to αEGFR mAb, as well as the combination (responder), while CXF233 was resistant to all treatments (non-responder).

### 3.3. [^18^F]FSPG PET of PDX Tumors

**Figure 3:**
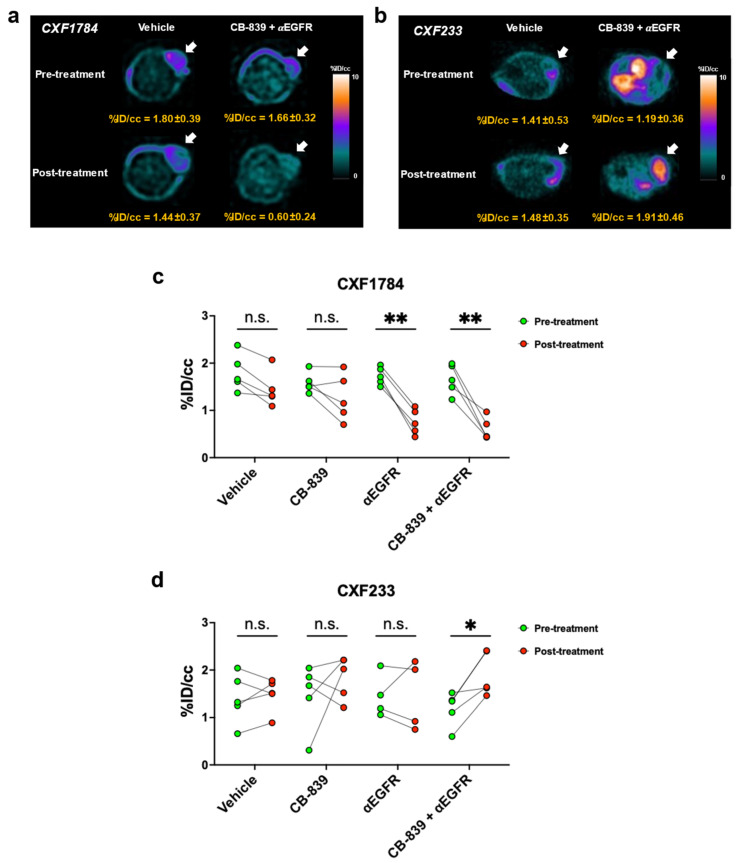
Preclinical [^18^F]FSPG PET predicts treatment efficacy. Representative [^18^F]FSPG-PET images of PDX murine models ((**a**): CXF1784, (**b**): CXF233). Quantitative analysis of PET images of PDX tumors ((**c**): CXF1784, (**d**): CXF233). Statistical significance is defined as follows: n.s. (not significant), * *p* < 0.05 and ** *p* < 0.001.

To explore the relationship between [^18^F]FSPG PET and tumor response to therapy, PDX-bearing mice were imaged immediately prior to and one week following initiation of treatment (Figure 3). We found decreased [^18^F]FSPG PET at one week relative to baseline correlated with future treatment efficacy and response. Similar to manifesting tumor volume changes, the CXF1784 case demonstrated decreased [^18^F]FSPG uptake of tumor in both the single agent αEGFR and combination groups (−56.8 ± 12.3%, *p* = 0.0002 and −62.8 ± 16.6%, *p* = 0.0003, respectively, Figure 3a,c). Likewise, the CXF233 case, which was considered non-responsive and progressing, failed to exhibit decreased PET with any treatment. In fact, combination treatment actually led to significantly elevated [^18^F]FSPG uptake in this tumor model (70.8 ± 50.1%, *p* = 0.025, Figure 3b,d). No significant change in [^18^F]FSPG uptake of tumor was observed in the CXF1972, which had relatively low baseline [^18^F]FSPG uptake (Figure S1b). Quantification of tumor to muscle ratio yielded similar results (Figure S2). This suggested that CRCs with poor initial [^18^F]FSPG uptake are not well-monitored with this approach. Interestingly, single agent glutaminase inhibition did not result in decreased [^18^F]FSPG, suggesting that EGFR inhibition was key determinate of [^18^F]FSPG-predicted response.

### 3.4. Histology

**Figure 4:**
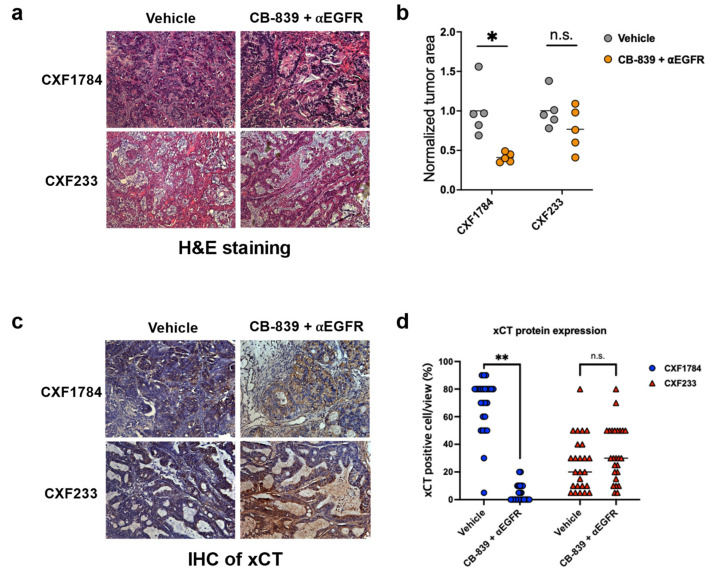
Histology. (**a**) Representative H&E images of tumor growth in PDX models (20× magnification). (**b**) Alive tumor ratio analysis results. Each tumor area was normalized by the mean area of the vehicle group for each PDX case. (**c**) Representative immunohistochemical staining results of xCT in PDX models (20× magnification). (**d**) Quantitative analysis of xCT IHC positivity (*n* = 5). Bar on dot plots indicates mean value and statistical significance is defined as follows: n.s. (not significant), * *p* < 0.01, and ** *p* < 0.0001.

To confirm the clinical responses observed, we further investigated the CXF1784 (responding) and CXF233 (non-responding) cases ex vivo using collected tissue samples following imaging and therapy. H&E staining demonstrated key morphology changes between vehicle and responsive treatment groups. Alive tumor cell ratio analysis using tumor areas normalized by mean tumor area of the vehicle group from H&E staining showed a significant decrease in the CXF1784 case (*p* = 0.0046) (Figure 4a,b and Table 2). In contrast, the CXF233 case showed no significant difference of tumor cell ratio or alive tumor area. Uptake of [^18^F]FSPG in tumor cells is related to protein expression of the xCT transporter, a glutamate–cystine exchanger (SLC7A11/SLC3A2 (4F2hc) heterodimer) that transports L-cystine (Cys-S-S-Cys) into the cell and L-glutamate to the extracellular compartment [16]. We carried out IHC to confirm xCT and CD44 protein expression, which has also been implicated in [^18^F]FSPG PET, in each case (Figure 4c and Figure S3). After the combined treatment, CXF233 showed higher xCT expression compared with the same vehicle-treated tumors, as well as vehicle- or combination-treated CXF1784 tumors (*p* < 0.0001, Figure 4d). We found that CD44 was only expressed in the CXF233 case and was independent of treatment, suggesting that CD44 was not a critical determinant of [^18^F]FSPG uptake in the CXF1784 model or affected by treatment in CXF233 (Figure S3).

## 4. Discussion

To our knowledge, this is the first preclinical study to evaluate [^18^F]FSPG PET to predict response to glutaminase and EGFR inhibition in CRC. Consistent with prior cell-line-based studies using xenograft models, [7] we found that certain, but not all, WT *KRAS* CRC PDXs were sensitive to this combination. Importantly, decreased [^18^F]FSPG PET following treatment was a hallmark of sensitivity suggesting a role for this PET tracer in clinical trials.

Tumor classification as a predictive motif of sensitivity to therapy is a critical aspect of patient-tailored care. Of note, patients with advanced WT *KRAS* CRC tend to experience limited benefit from EGFR-targeted therapy [17,18]. To select diverse yet representative models for evaluating [^18^F]FSPG PET in this setting, we leveraged the CMS classification [14]. As reported, the CMS1 group is associated with decreased survival after relapse [19]. A recent clinical study demonstrated that CMS1 had poorer survival than CMS2-4 when challenged with chemotherapy, whereas WT *KRAS* CMS2 patients received more benefit from EGFR-targeted therapy [20]. Consistent with these previous reports, we found that the CXF1784 PDX (CMS2) was responsive to single EGFR-targeted therapy, as well as the combination. In contrast, the CXF233 PDX tumor (CMS1) subtype showed limited response to any treatments tested here. As further evidence, CXF233 carries *ERBB3*, *ERBB4*, and *PIK3CA* mutations, which is consistent with the CMS1 subtype and may also contribute to the lack of response with inhibition of these therapeutic axes [21,22,23]. Importantly, regardless of classification, [^18^F]FSPG PET accurately predicted response among these models.

The xCT transporter is not only correlated with [^18^F]FSPG uptake [16], but it also plays a critical role in tumor invasion, metastasis, and poor prognosis in multiple malignancies [24]. Expression of xCT is regulated by multifaceted factors. Genetically, the tumor suppressor gene *TP53* may reduce cystine uptake and promote ferroptosis, a form of non-apoptotic cell death involving the catastrophic accumulation of lipid peroxidation and ROS [25]. Liu et al. demonstrated that mutant *TP53* could bind to nuclear factor erythroid 2-related factor (NRF2) and impairs the function of NRF2 to maintain xCT expression [26]. Several studies reported that the expression of xCT is regulated by NRF2 [27,28,29]. Similar to the interaction between CD44v and xCT [30], *EGFR* is associated with xCT and its stabilization on the plasma membrane to facilitate cystine uptake in glioma cells [31]. Although both CXF233 and CXF1784 had a *TP53* mutation, CXF233 also showed mutations of *NFE2L2* (encodes *NRF2*) and *EGFR*, along with higher protein expressions of xCT and CD44. While CD44 was not an obvious correlate of [^18^F]FSPG PET in this study, a combination of genetic alterations may contribute to the durable [^18^F]FSPG uptake following treatment in non-responding tumors such as CXF233. As such, complementary to potentially confounding molecular features, additional phenotypic characteristics such as [^18^F]FSPG uptake could be applied towards a mixed model classification useful to identify patients likely to respond to therapy at early timepoints.

In this study, we did not evaluate the role of vascularity, which could affect [^18^F]FSPG uptake, particularly at early time points following administration and before steady state was reached. Furthermore, this study featured female tumor-bearing hosts. It is not known whether [^18^F]FSPG accumulation profiles are dependent upon the sex of the host within the context of PDX imaging studies [32]. Therefore, additional comprehensive in vivo assessments may provide better understanding of this relationship.

## 5. Conclusions

The current findings reported here with [^18^F]FSPG warrant further investigation using a larger sample size and featuring additional CRC models with genetic complexity. Moreover, further information on the comprehensive metabolomics and genetics of tumors related to oxidative stress may provide further insights towards the underlying tumor biology and chemoresistance mechanisms that portend [^18^F]FSPG accumulation. Nonetheless, our findings suggest feasibility of [^18^F]FSPG PET as an early imaging signature of response to EGFR and glutaminase inhibition in the WT *KRAS* CRC setting. The utility of [^18^F]FSPG PET in imaging tumors beyond colorectal cancer and in monitoring treatment response should be the emphasis of future studies.

## Figures and Tables

**Table 1:** Clinicopathological characteristics of the donor patients.

PDX ID	CXF233	CXF1784	CXF1972	CXF1753
Patient information				
Gender	Female	Male	Male	Male
Age	74	65	62	61
Histology	Adenocarcinoma	Adenocarcinoma	Adenocarcinoma	Adenocarcinoma
Stage at surgery	TxN2M0	M1 liver	N/A	T3N1M1
Chemotherapy prior to surgery	No	FolFOX	not known	not known
Origin of xenograft	Primary (Colon)	Metastasis (Liver)	Metastasis (Pleura)	Metastasis (Peritoneum)

**Table 2:** Results of alive tumor ratio analysis.

PDX ID	Group	Mouse ID	Tumor Area (mm^2^)	Normalized Tumor Area *	Alive Tumor Area (%)
CXF1784	Vehicle	V1	32.75	0.69	69.20%
		V2	45.82	0.96	47.39%
		V3	46.48	0.97	43.39%
		V4	74.48	1.56	40.11%
		V5	39.37	0.82	20.15%
	CB-839 + 𝛼EGFR	CB + P1	17.01	0.36	61.39%
		CB + P2	16.75	0.35	58.41%
		CB + P3	21.74	0.45	66.66%
		CB + P4	23.63	0.49	71.28%
		CB + P5	19.34	0.4	58.00%
CXF233	Vehicle	V6	65.82	0.89	33.28%
		V7	101.79	1.38	48.53%
		V8	69.99	0.95	55.25%
		V9	57.45	0.78	14.34%
		V10	74.95	1.01	20.69%
	CB-839 + 𝛼EGFR	CB + CTX1	72.72	0.98	33.15%
		CB + CTX2	30.64	0.41	23.30%
		CB + CTX3	56.56	0.76	15.47%
		CB + CTX4	80.67	1.09	16.64%
		CB + CTX5	44.46	0.6	21.23%

* Each tumor area was normalized by mean tumor area of vehicle group.

## Data Availability

The datasets generated and/or analyzed during the current study are available from the corresponding author upon reasonable request.

## Supplementary Materials

The following supporting information can be downloaded at: https://www.mdpi.com/article/10.3390/tomography9020041/s1. Figure S1. Combined CB-839/αEGFR mAb treatment. (a) PDX murine model was treated with either 𝛼EGFR monoclonal antibody (mAb), CB-839, or CB-839 and 𝛼EGFR mAb. Error bars represent ± standard deviation (SD) (*n* = 5). (b) Quantitative analysis of PET images of CXF1972 PDX tumor. Figure S2. Tumor-to-muscle ratio of [18F]FSPG PET uptake. Quantitative analysis of PET images of PDX tumors ((a): CXF1784, (b): CXF233, (c): CXF1972). %ID/cc values of muscles in the flank region were also obtained and tumor-to-muscle (T/M) uptake ratio was calculated. Statistical significance is defined as follows: n.s. (not significant), * *p* < 0.05 and ** *p* < 0.001. Figure S3. IHC of CD44. Representative immunohistochemical staining results of CD44 in PDX models (20× magnification). CD44 was expressed in all CXF233 samples at a comparable level while no detectable CD44 expression was observed in the CXF1784 case.
