# Supplementary material for: Feasibility of [18F]FSPG PET for Early Response Assessment to Combined Blockade of EGFR and Glutamine Metabolism in Wild-Type KRAS Colorectal Cancer
Source: Tomography. 2023 Feb 24;9(2):497–508. doi: 10.3390/tomography9020041 (PMC10037609; doi:10.3390/tomography9020041)
Supplement: Supplementary file 1 [file tomography-09-00041-s001.zip › tomography-2223443-supplementary.pdf]

# Supplementary Materials

## Supplementary Figure S1

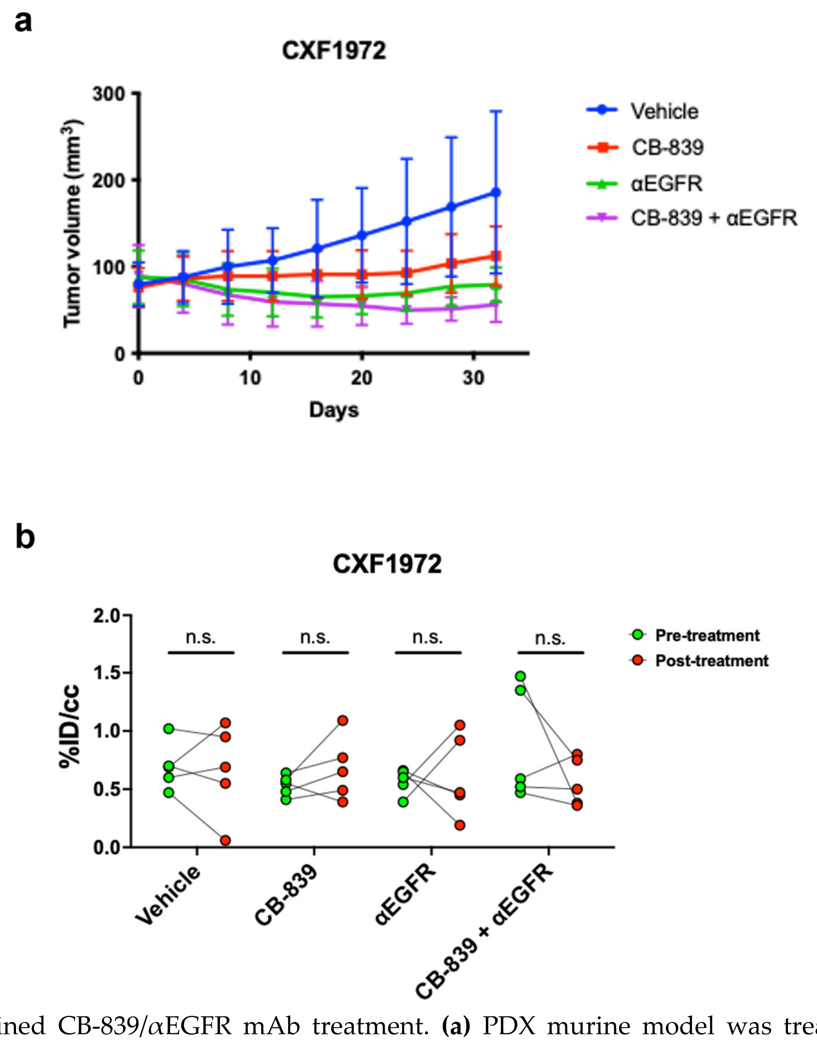


**Figure S1.** Combined CB-839/*α*EGFR mAb treatment. **(a)** PDX murine model was treated with either *α*EGFR monoclonal antibody (mAb), CB-839, or CB-839 and *α*EGFR mAb. Error bars represent ± standard deviation (SD) ($n$ = 5). **(b)** Quantitative analysis of PET images of CXF1972 PDX tumor.

# Supplementary Figure S2

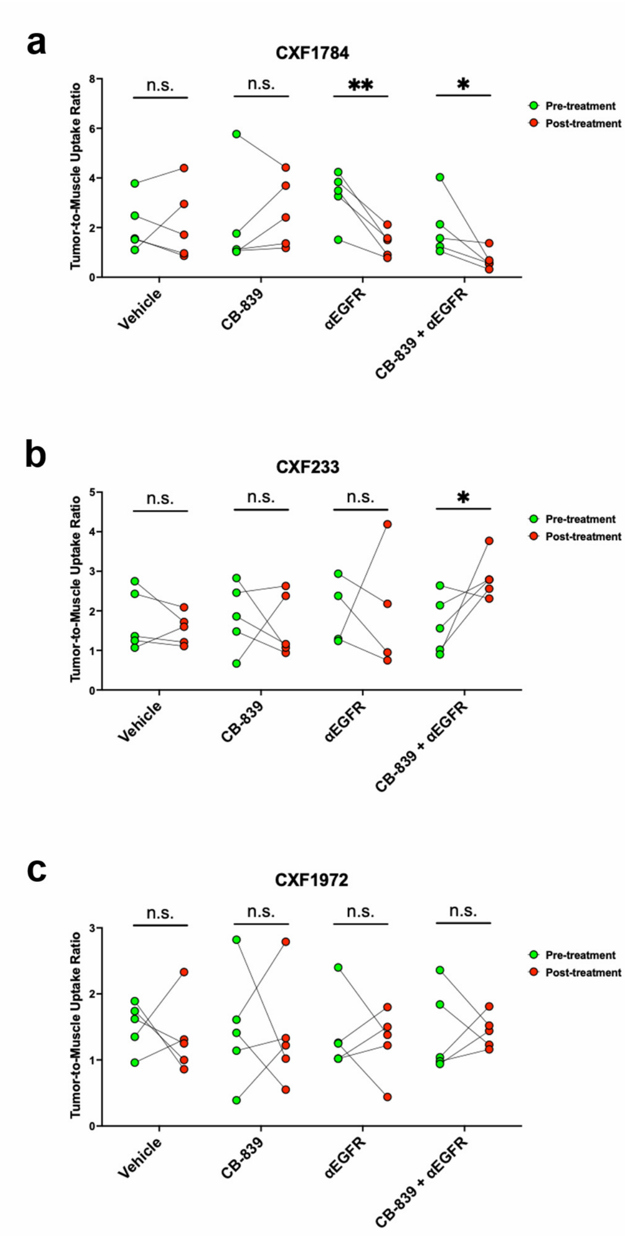


**Figure S2.** Tumor-to-muscle ratio of [$^{18}$F]FSPG PET uptake. Quantitative analysis of PET images of PDX tumors (**a**: CXF1784, **b**: CXF233, **c**: CXF1972). %ID/cc values of muscles in the flank region were also obtained and tumor-to-muscle (T/M) uptake ratio was calculated. Statistical significance is defined as follows: n.s. (not significant), * $p < 0.05$ and ** $p < 0.001$.

## Supplementary Figure S3

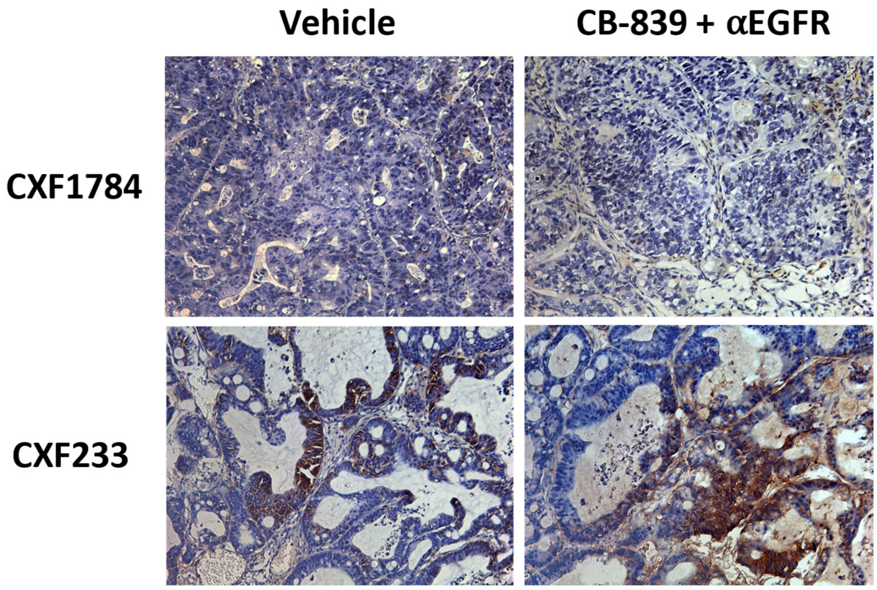


**Figure S3.** IHC of CD44. Representative immunohistochemical staining results of CD44 in PDX models (20X magnification). CD44 was expressed in all CXF233 samples at a comparable level while no detectable CD44 expression was observed in the CXF1784 case.
